# Inhibition of cGAS–STING pathway alleviates neuroinflammation-induced retinal ganglion cell death after ischemia/reperfusion injury

**DOI:** 10.1038/s41419-023-06140-0

**Published:** 2023-09-19

**Authors:** Xingdi Wu, Naiji Yu, Zifan Ye, Yuxiang Gu, Chengshou Zhang, Min Chen, Kaijun Wang

**Affiliations:** 1 Eye Center of the Second Affiliated Hospital, School of Medicine, Zhejiang University, Hangzhou, Zhejiang Province China; 2 Zhejiang Provincial Key Lab of Ophthalmology, Hangzhou, Zhejiang Province China; 3 Department of Ophthalmology, The First People’s Hospital of Xiaoshan District, Hangzhou, Zhejiang Province China

**Keywords:** Neurodegeneration, Inflammation, Medical research

## Abstract

Acute glaucoma is a vision-threatening disease characterized by a sudden elevation in intraocular pressure (IOP), followed by retinal ganglion cell (RGC) death. Cytosolic double-stranded DNA (dsDNA)—a damage-associated molecular pattern (DAMP) that triggers inflammation and immune responses—has been implicated in the pathogenesis of IOP-induced RGC death, but the underlying mechanism is not entirely clear. In this study, we investigated the effect of the inflammatory cascade on dsDNA recognition and examined the neuroprotective effect of the cyclic GMP-AMP (cGAMP) synthase (cGAS) antagonist A151 on a retinal ischemia/reperfusion (RIR) mouse model. Our findings reveal a novel mechanism of microglia-induced neuroinflammation-mediated RGC death associated with glaucomatous vision loss. We found that RIR injury facilitated the release of dsDNA, which initiated inflammatory responses by activating cGAS–stimulator of interferon genes (STING) pathway. Correspondingly, elevated expressions of cGAS and STING were found in retinal samples from human glaucoma donors. Furthermore, we found that deletion or inhibition of cGAS or STING in microglia transfected with poly(dA:dT) specifically decreased microglia activation and inflammation response. We also observed that A151 treatment promoted poly(dA:dT)--stimulated changes in polarization from the M1 to the M2 phenotype in microglia. Subsequently, A151 administered to mice effectively inhibited the cGAS–STING pathway, absent in melanoma 2 (AIM2) inflammasome and pyroptosis-related molecules. Furthermore, A151 administration significantly reduced neuroinflammation, ameliorated RGC death and RGC-related reductions in visual function. These findings provide a unique perspective on glaucomatous neuropathogenesis and suggest cGAS as an underlying target of retinal inflammation to provide a potential therapeutic for acute glaucoma.

## Introduction

Acute glaucoma, one of the most common types of glaucoma in Asia, is characterized by a sudden and dramatic increase in intraocular pressure (IOP), leading to retinal ischemia/reperfusion (RIR) injury and RGC death [1–3]. Although the factors influencing the loss of RGCs have not been fully elucidated, inflammation within the retina is closely associated with optic nerve damage and neurodegeneration [4]. Importantly, microglia play a key role in neuroinflammation, which is a key process in glaucoma [5–7]. Ischemia-induced retinal damage is likely to be irreversible; even with readily available pharmacological and surgical treatment, it can still cause permanent visual impairment in many patients [8]. Persistent postischemic inflammation may mediate ongoing neuronal immunologic deterioration and amplify RGC death [9]. RIR not only directly induces the death of RGCs, but also triggers the damage-associated molecular pattern (DAMP)-dependent neuroinflammation that activates microglia, further inducing RGC death [10, 11]. The cytosolic double-stranded DNA (dsDNA) released from necrotic RGCs after an episode of retinal ischemia is a potential DAMP, and the mechanisms underlying the recognition of dsDNA by nucleic acid-sensing cyclic GMP-AMP (cGAMP) synthase (cGAS) in ischemic retinal inflammation have not yet been explored.

In recent years, the cGAS has attracted attention as an important sensor for cytosolic detection and the recognition of exogenous and endogenous cytosolic DNA [12, 13]. Upon binding directly to DNA, cGAS triggers the production of cyclic dinucleotide 2’-3’-cGAMP, which binds to and activates stimulator of interferon genes (STING). This leads to the production of type I interferons (IFNs) via the transcription factor interferon regulatory factor 3 (IRF3) and the expression of nuclear factor-κB (NF-κB)-dependent pro-inflammatory cytokines [13–15]. Overstimulation of the cGAS–STING pathway can lead to the death of neurons [16, 17], which is implicated in many neurological diseases. Previous studies have shown that the accumulation of cytosolic dsDNA derived from necrotic neuronal cells in ischemic stroke activates the cGAS–STING pathway and other inflammatory cascades [18]. Notably, recent in vivo studies have suggested that cGAS–STING pathway activation is not only a side effect of injury, but also actively promotes apoptosis [19]. Therefore, targeting cGAS–STING signaling may be a reasonable approach for controlling cytosolic dsDNA-induced inflammatory responses and ameliorating associated pathologies.

Studies have highlighted the role of signal transduction in a caspase-dependent inflammasome pathway in RIR injury [7, 20]. AIM2 is a key sensor for detecting the presence of cytosolic dsDNA. The activation of AIM2 initiates inflammasome assembly, which is involved in various sterile self-DNA-triggered inflammatory conditions. This prompts the maturation and secretion of the IL-1β and IL-18 cytokines, as well as pyroptosis (a pro-inflammatory form of cell death) [21]. In addition to the post-translational AIM2 inflammasome assembly triggered directly by cytosolic dsDNA, type I interferons stimulated by the cGAS–STING pathway can cooperate with AIM2 inflammasomes to orchestrate immune responses, further demonstrating that cytosolic dsDNA is involved in multiple parallel pathways that, together, maximize inflammatory responses [22].

In most neurodegenerative diseases, inhibition of the cGAS–STING pathway provides an avenue for therapeutic intervention [17]. The retina is an extension of the central nervous system (CNS); therefore, targeting the cGAS–STING pathway may contribute to the discovery of new approaches to acute glaucoma. ODN TTAGGG, also known as A151, is a synthetic oligonucleotide comprising the immunosuppressive TTAGGG motif, and it is a novel inhibitor of cGAS and AIM2 by competing with DNA [23, 24]. However, it is uncertain whether using A151 to pharmacologically antagonize dsDNA-sensing cGAS and the AIM2 inflammasome can mediate neuroinflammation and protect RGCs from RIR injury.

## Results

### Upregulation of dsDNA and dsDNA sensors triggered by RIR injury

**Fig. 1:**
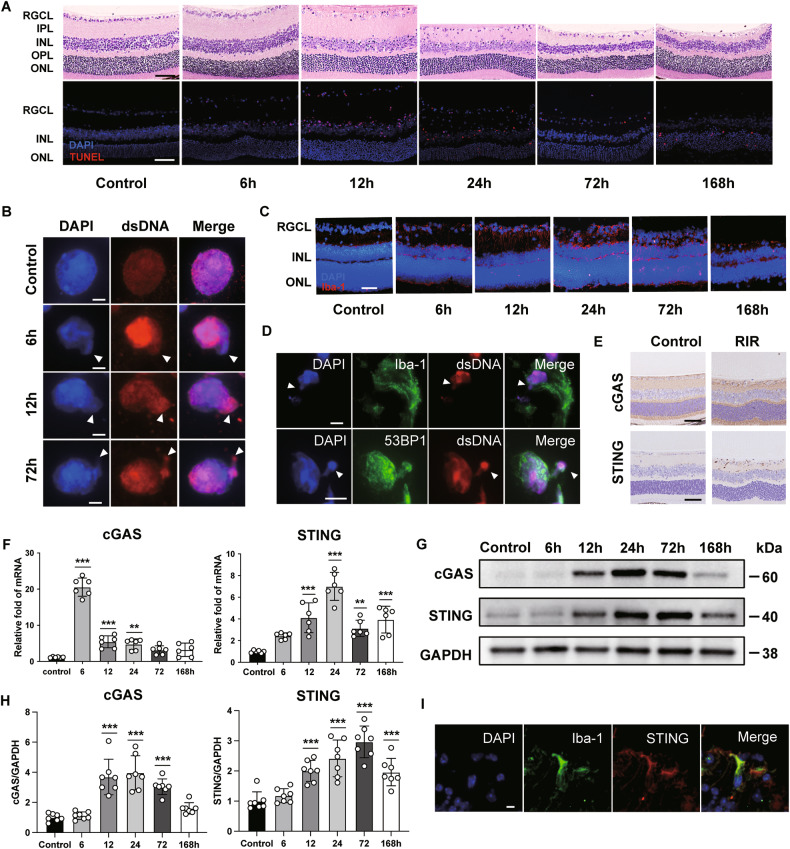
Accumulation of dsDNA and upregulation of cGAS after retinal ischemic injury. **A** Upper row: HE staining of the retinas at different times after RIR injury; Lower row: Representative images of TUNEL-positive cells in the retinas at different times after RIR injury. Scale bar: 50 μm. **B** Representative immunofluorescent images of dsDNA in the RGC at 6, 12, and 72 h after RIR injury as well as in the control retinas; arrowheads indicate disintegration of the nucleus. Scale bar: 5 μm. **C** Representative images of microglia cells in the retinas at different times after RIR injury. Scale bar: 50 μm. **D** Representative images of double immunofluorescent staining of 53BP1, dsDNA (d upper row), Iba-1, and dsDNA (d lower row); arrowheads indicate cytoplasmic dsDNA. Scale bar: 5 μm. **E** Immunohistochemistry staining of cGAS and STING 3 d after IR injury, as well as in the control group. Scale bar: 50 μM. **F** The mRNA expression levels of cGAS and STING after RIR injury (*n* = 6). **G**, **H** Western blot and quantitative analyses of cGAS and STING in the retinas at different times after RIR injury (*n* = 7). **I** Representative images of double immunofluorescent staining of Iba-1 and STING. Scale bar: 5 μm. Data are shown as means ± SEM. The dots represent biological replicates. **P < 0.01, ***P < 0.001. One-way ANOVA followed by a Bonferroni post hoc test. RGCL retinal ganglion cell layer (RGCL), IPL inner plexiform layer, INL inner nuclear layer, OPL outer plexiform layer, ONL outer nuclear layer.

In a mouse model of RIR injury, the retinas suffered morphological alterations and RGC death, which led to irreversible damage to RGC (Fig. 1A). We also found that neuroinflammation was most pronounced in the early stage after reperfusion and persisted for at least 7 d (Supplementary Fig. S1). Notably, dsDNA is a potent DAMP of neuroinflammation following RIR injury. To detect the expression of this DAMP molecule in the retinas, we examined dsDNA with immunofluorescent staining after inducing RIR. We found that the intensities of dsDNA increased with a nucleoplasmic relocation in the RGC layer (RGCL), and nucleus disintegration was also detected 6, 12, and 72 h after RIR (Fig. 1B). Microglia are representative immune cells that quickly respond to RIR injury. Thus, we observed significant activation of microglia, characterized by typical morphological changes, that promoted the expression of cell-type-specific markers for microglia (Iba-1) (Fig. 1C). After co-staining with dsDNA, we found cytosolic dsDNA in microglial cells. Moreover, following RIR injury, most of the dsDNA staining appeared to be nuclear, with close spatial colocalization with DAPI and 53BP1—a key player in DNA damage response. Cytoplasmic dsDNA was also detected in the retinas subjected to RIR injury (Fig. 1D).

cGAS is a key cytosolic dsDNA sensor. Paralleling the heightened deposition of dsDNA, we found that Immunohistochemistry (IHC) revealed an elevation of cGAS and STING levels in retinas 72 h after RIR injury (Fig. 1E). We also found that mRNA levels of cGAS and STING were abundantly upregulated at 6 h and 24 h, respectively, after RIR injury (Fig. 1F). The protein levels of cGAS and STING were also upregulated at 24 h and 72 h, respectively (Fig. 1G, H). The time-dependent upregulation of cGAS and STING in the retinas was associated with RGC damage and microglia activation. Aside from dsDNA, STING was also mainly expressed in Iba-1-positive cells, indicating a strong relationship between the cGAS–STING pathway and microglia activation (Fig. 1I). Taken together, these data suggest that increased release of self-derived dsDNA within the cytosol following RIR injury may trigger cGAS–STING pathway activation and a concurrent activation of microglia.

### The DNA sensor cGAS participates in microglia-mediated neuroinflammation

**Fig. 2:**
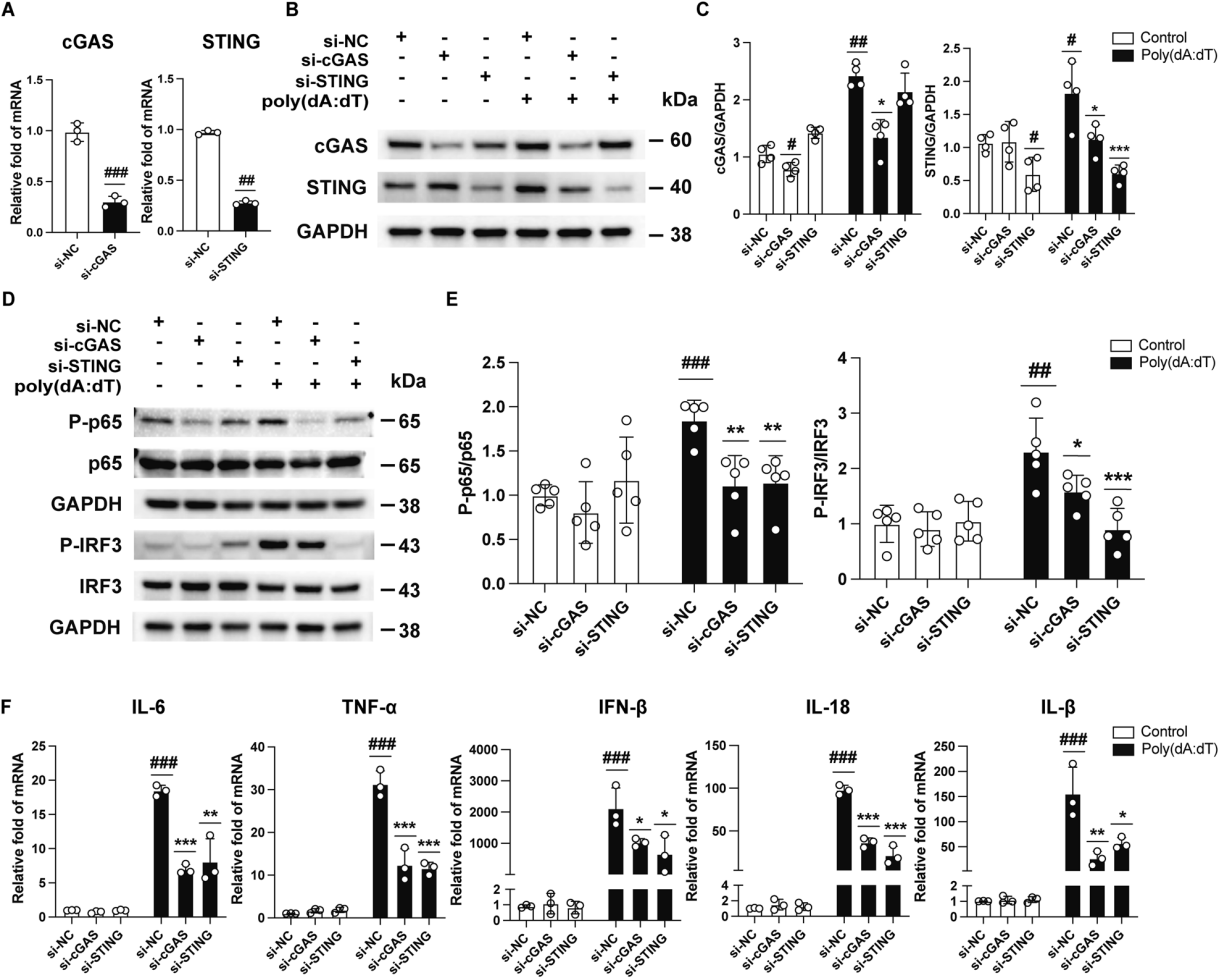
Knockdown of cGAS or STING in BV2 cells attenuates poly(dA:dT)-induced cGAS activation and inflammation in vitro. **A** The mRNA expression levels of cGAS or STING after transfection with siRNA against cGAS or STING (*n* = 3). **B**, **C** Western blot and quantitative analyses of cGAS and STING in cGAS- or STING-silenced BV2 cells after transfection with poly(dA:dT) (*n* = 4). **D**, **E** Western blot and quantitative analyses of P-IRF3, IRF3, P-p65, and p65 in cGAS- or STING-silenced BV2 cells after transfection with poly(dA:dT) (*n* = 5). **F** The mRNA expression levels of IL-6, TNF-α, IFN-β, IL-18, and IL-1β (*n* = 3). Data are shown as means ± SEM. The dots represent biological replicates. ^#^*P* < 0.05, ^##^*P* < 0.01, ^###^*P* < 0.001 compared with the si-NC group. *P < .005, **P < 0.01, ***P < 0.001 compared with the si–NC + poly(dA:dT) group. One-way ANOVA followed by a Bonferroni post hoc test and a two-tailed Student’s *t* test.

Although microglia-mediated inflammation participates in RIR injury, the innate immune pathways activated in microglia following RIR injury remain ambiguous. To check whether cGAS sensors are involved in microglia, we confirmed the role of cGAS and STING in BV2 microglial cell lines using siRNAs. First, siRNAs targeting cGAS or STING significantly inhibited the mRNA and protein levels of cGAS or STING, respectively (Fig. 2A, B). To further investigate the activation of dsDNA-sensing cGAS in microglia with stimulation of dsDNA, a BV2 microglial cell line was transfected with poly(dA:dT)—a synthetic analogue of dsDNA—in vitro for comparative analysis. As expected, cGAS and STING knockdown significantly downregulated poly(dA:dT)-induced cGAS and STING activation (Fig. 2B, C). In addition, poly(dA:dT)-induced IRF3 and p65 phosphorylation were also downregulated (Fig. 2D, E) in both the si-cGAS and si-STING groups compared to si-NC group. Since IRF3 and p65 are major molecular bridges between cGAS–STING pathway activation and DNA-driven immune response, which, respectively mediate type I IFNs and inflammatory cytokines, those downstream cytokines were detected [13]. Poly(dA:dT) greatly increased, whereas the knockdown of cGAS reduced the transcriptional upregulation of cytokines, including IL-6, TNF-α, IFN-β, IL-18, and IL-1β, similar to the effect of STING knockdown (Fig. 2F). Together, these results suggest that cGAS is a DNA sensor that mediates the activation of the type I interferon and NF-κB pathways by sensing DAMP (e.g., dsDNA) in microglia.

### A151 attenuates poly(dA:dT)-induced cGAS activation and pyroptosis in vitro

**Fig. 3:**
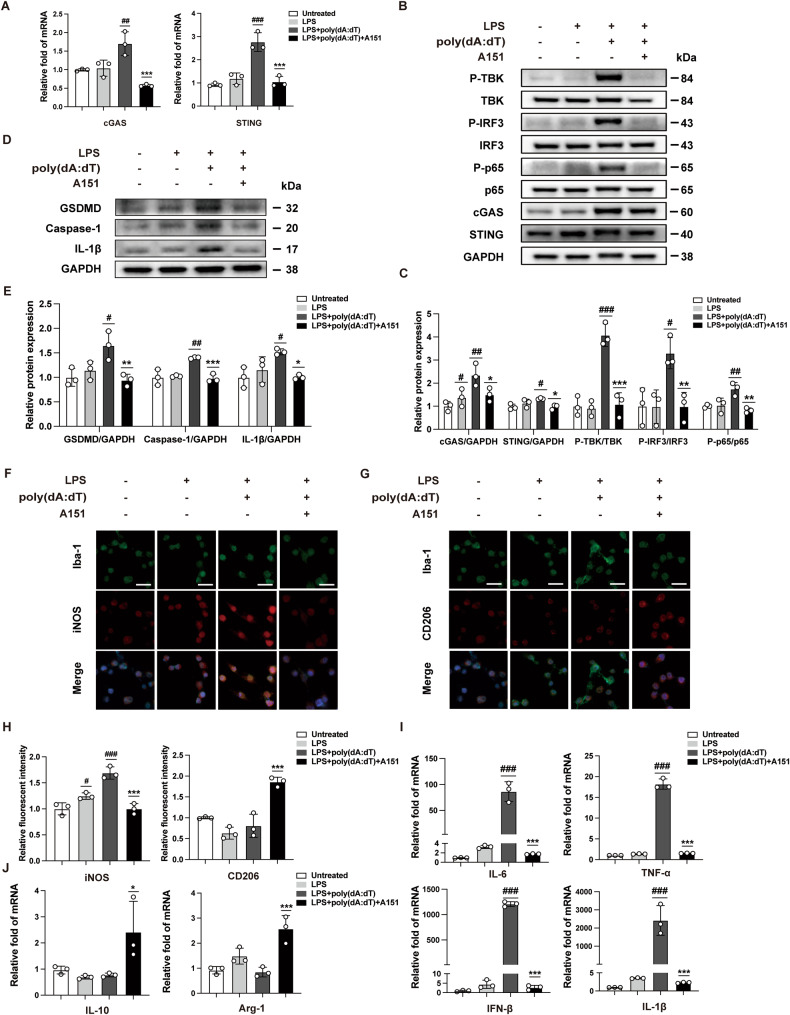
A151 alleviates cGAS–STING pathway activity, pyroptosis-associated proteins in vitro. **A** The mRNA levels of cGAS or STING were determined using qRT–PCR (*n* = 3). **B**, **C** Western blot and quantitative analyses of cGAS, STING and phosphorylation of TBK1, p65 and IRF3 in BV2 cells (*n* = 3). **D**, **E** Western blot and quantitative analyses of GSDMD, Caspase-1 and IL-1β in BV2 cells (*n* = 3). **F**, **G** Immunofluorescence was conducted to detect Iba-1, iNOS and CD206 expression levels in BV2 cells. Scale bar: 20 μm. **H** Quantitative analysis of iNOS and CD206 (*n* = 3). **I**, **J** The mRNA levels of pro-inflammatory and anti-inflammatory cytokines, including IL-6, TNF-α, IL-1β, IFN-β, IL-10 and Arg-1 (*n* = 3) Data are shown as means ± SEM. The dots represent biological replicates. ^#^*P* < 0.05, ^##^*P* < 0.01, ^###^*P* < 0.001 compared with the untreated group. *P < 0.05, **P < 0.01, ***P < 0.001 compared with the LPS + poly(dA:dT) group. One-way ANOVA followed by a Bonferroni post hoc test.

To address how the cGAS–STING pathway contributes to neuroinflammation following RIR injury, we evaluated BV2 microglial cell lines transfected with poly(dA:dT) combined with A151 treatment. A151 effectively abrogates cGAS activation in response to the accumulation of cytosolic DNA competitively with DNA [23]. Firstly, The cytotoxicity of the A151 was evaluated using Calcein-AM/ propidium iodide (PI) and Cell Counting Kit-8 (CCK-8) assays (Supplementary Fig. S2A, B). The BV2 cells were treated with A151 or PBS. Based on the live/dead cell staining, the majority of BV2 cells treated with A151 displayed green fluorescence. Accordingly, CCK-8 values were normalized to PBS-treated controls and showed no difference between the two groups. Subsequently, we found elevated transcription of cGAS and STING in LPS-primed BV2 cells stimulated with poly(dA:dT) compared with the blank control group, whereas the elevation of these mRNA transcripts was directly suppressed by A151 (Fig. 3A), as well as the protein level was also suppressed by A151 (Fig. 3B, C). Moreover, core proteins of these transcripts were proved paralleled. cGAS–STING pathway activation—as estimated by the phosphorylation rates of the downstream molecules TBK1, IRF3, and NF-κB p65—was also enhanced by exposure to poly(dA:dT) and inhibited by A151 (Fig. 3B, C). We also observed that protein levels of pyroptosis-associated proteins (Caspase-1, IL-1β, and GSDMD) were elevated in microglia stimulated with poly (dA:dT), whereas A151 markedly repressed this alterations (Fig. 3D, E). The cGAS–STING pathway activation and Caspase-1 induced pyroptosis were further confirmed by immunofluorescence (Supplementary Fig. S3A, B). M1 microglia are known to release inflammatory cytokines, while M2 microglia conventionally release anti-inflammatory cytokines [25]. Accordingly, A151 reversed the increase in inducible nitric oxide synthase (iNOS) (M1 marker) and the decrease in CD206 (M2 marker) induced by poly(dA:dT) (Fig. 3F, G, H). To confirm this result, the mRNA expression of microglia markers, including IL-1β, IL-6, TNF-α, Arg-1 and IL-10, were measured by using qRT–PCR (Fig. 3I, J). Taken together, these results suggested that pharmacological inhibition of dsDNA-sensing cGAS by A151 could ameliorate cGAS–STING pathway activation and Caspase-1 induced pyroptosis. And it could also exert anti-inflammatory activity by modulating microglia polarization, and subsequent regulating inflammation cytokines expression.

### Inhibition of microglial activation and cGAS signaling by A151 after RIR injury

**Fig. 4:**
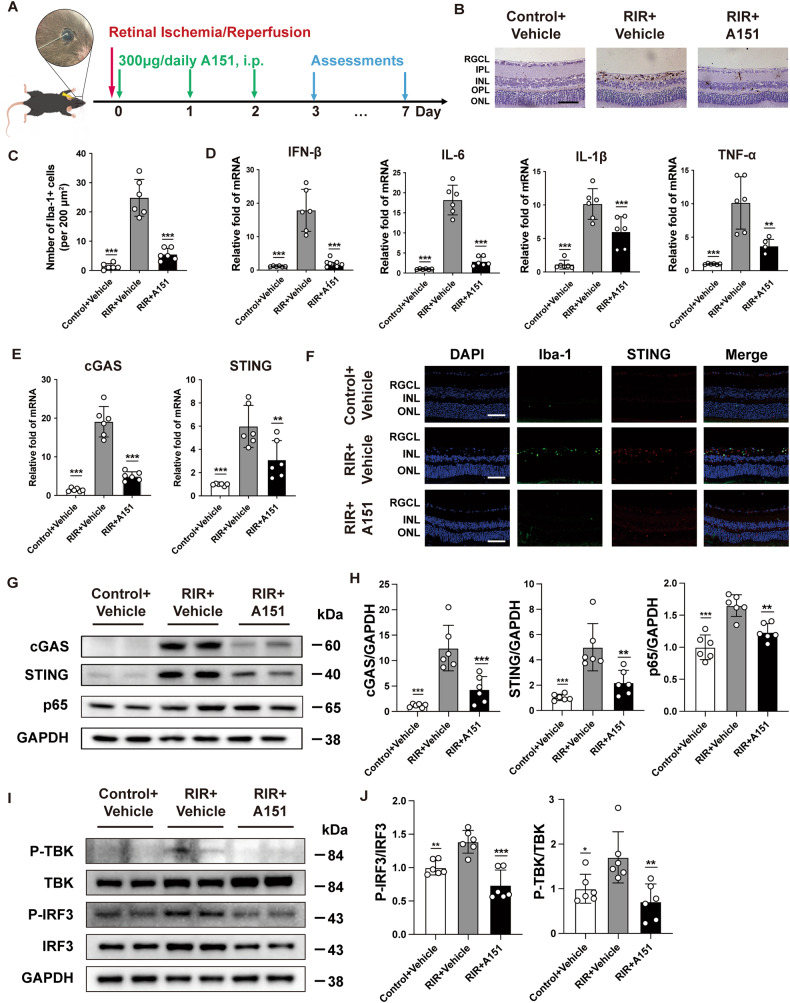
A151 inhibits the RIR-induced expression of cGAS signaling and inflammatory cytokines. **A** Flowchart illustrating the administration and experimental design of A151. Mice received daily intraperitoneal (IP) injections of A151 (300 μg) or an equal volume of phosphate-buffered saline (PBS) vehicle for 3 consecutive days following RIR injury. **B** Immunohistochemistry staining of microglia cells in the retinas of the various groups. Microglia cells were marked with Iba-1. Scale bar: 50 μm. **C** Quantification of Iba-1-positive cells in the retina (*n* = 6). **D** The mRNA expression levels of inflammatory cytokines, including IL-6, TNF-α, IFN-β, and IL-1β, were determined using qRT–PCR (*n* = 6). **E** The mRNA expression levels of inflammatory cytokines, including cGAS, STING were determined using qRT–PCR (*n* = 6). **F** Representative images of double immunofluorescent staining of Iba-1 and STING. Scale bar: 50 μm. **G**, **H** Western blot and quantitative analyses of p65, cGAS, and STING in the retinas of the various groups (*n* = 6). **I**, **J** Western blot and quantitative analyses of phosphorylation of TBK1 and IRF3 in the retinas of the various groups (*n* = 6). Data are shown as means ± SEM. The dots represent biological replicates. *P < 0.05, **P < 0.01, ***P < 0.001 compared with the RIR + vehicle group. One-way ANOVA followed by a Bonferroni post hoc test. RGCL retinal ganglion cell layer, INL inner nuclear layer, ONL outer nuclear layer.

Considering the RIR-induced cGAS overexpression, inhibiting cGAS may be a logical method of protecting against the effects of RIR injury. The in vivo biocompatibility of A151 was evaluated in healthy mice first. Hematoxylin-eosin (HE) staining of the major visceral organs and retina of the A151 group did not exhibit noticeable histological changes compared to the PBS group (Supplementary Fig. S4A). The effect of A151 on cGAS signaling was examined in the retinas of RIR mice. A schematic diagram of the experimental protocol is shown in Fig. 4A. Retinas were evaluated 3 d after RIR injury. Immunohistochemistry staining revealed that the A151 treatment inhibited microglia activation after RIR injury (Fig. 4B, C). Transcription levels of inflammatory cytokines, including IL-6, TNF-α, IFN-β, and IL-1β, were highly upregulated in the retinas of vehicle-treated RIR mice, but A151 treatment reduced the expression of inflammatory cytokines (Fig. 4D). qRT–PCR revealed that cGAS and STING were greatly induced in the retinas after RIR injury, whereas A151 treatment abrogated the induction of both cGAS and STING (Fig. 4E). Western blot analysis further confirmed that the upregulated cGAS and STING (Fig. 4G, H), along with increased downstream NF-κB and phosphorylation of TBK1 and IRF3, were significantly attenuated by the administration of A151 (Fig. 4I, J). In concert with the above results, parallel alterations of STING and Iba-1 in diverse groups were also proved by immunofluorescence staining (Fig. 4F). These results suggest activation of the cGAS–STING pathway and microglia-mediated neuroinflammation in the mouse retinas after RIR injury, which was effectively suppressed by A151.

### Inhibition of the AIM2 inflammasome and pyroptosis by A151 after RIR injury

**Fig. 5:**
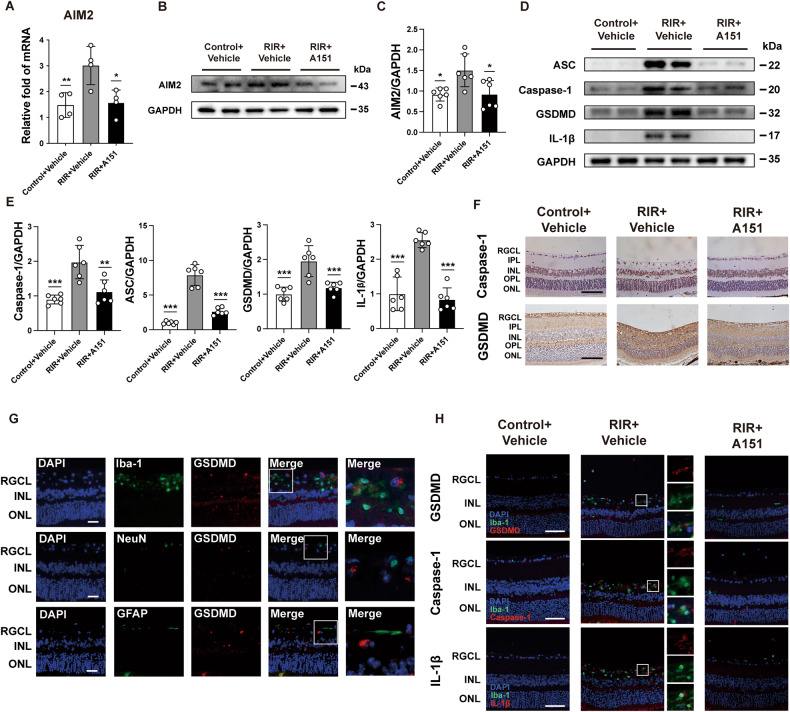
A151 inhibits the RIR-induced expression of Aim2 and pyroptosis. **A** The mRNA expression levels of AIM2 (*n* = 4). **B**, **C** Western blot and quantitative analyses of AIM2 in the retinas of the various groups (*n* = 6). **D**, **E** Western blot and quantitative analyses of ASC, Caspase-1, GSDMD, and IL-1β in the retinas of the various groups (*n* = 6). **F** Immunohistochemical staining of GSDMD and Caspase-1 in the retinas of the various groups. Scale bar: 50 μm. **G** Representative images of double immunofluorescent staining for Iba-1 and GSDMD, NeuN and GSDMD and for GFAP and GSDMD, in the retinas 3 d after RIR injury. Scale bar: 50 μm. **H** Representative images of double immunofluorescent staining of Iba-1 and GSDMD, Iba-1 and caspase-1, and Iba-1 and IL-1β in the control + vehicle, RIR + vehicle, and RIR + A151 groups. Scale bar: 50 μm. Data are shown as means ± SEM. The dots represent biological replicates. *P < 0.05, **P < 0.01, ***P < 0.001 compared with the RIR + vehicle group. One-way ANOVA followed by a Bonferroni post hoc test. RGCL retinal ganglion cell layer, IPL inner plexiform layer, INL inner nuclear layer, OPL outer plexiform layer, ONL outer nuclear layer.

AIM2 is another major player in the response to cytoplasmic dsDNA in sterile inflammation. However, it is unclear whether the A151 treatment can influence AIM2 activation and pyroptosis. Thus, the expression of the key AIM2-related proteins was examined. Western blot analysis demonstrated that the expression of AIM2 inflammasome-related molecules (AIM2/Caspase-1/ASC) in the retinas was upregulated at different time points during reperfusion. Notably, AIM2, Caspase-1, and ASC showed remarkable elevation that peaked at 24 h or 72 h. Meanwhile, GSDMD and IL-1β also increased progressively over time (Supplementary Fig. S5A, B). We also observed that A151 administration downregulated the expression of AIM2 in the retinas 72 h after RIR injury (Fig. 5A–C). In addition, pronounced increases in ASC, caspase-1, IL-1β, and GSDMD protein levels were observed in the RIR + vehicle group compared with the control group and were markedly suppressed by A151 (Fig. 5D, E). Meanwhile, changes in GSDMD and Caspase-1 were also proved through immunohistochemical analysis (Fig. 5F). Moreover, cell-type-specific analysis of GSDMD expression in the ischemic retinas revealed that GSDMD immunofluorescence signals were colocalized with microglial Iba-1 but rarely with NeuN and GFAP at 72 h after RIR injury (Fig. 5G). We also detected pyroptotic cell death in microglia subjected to ischemic injury. In addition, immunofluorescent staining of GSDMD, Caspase-1, and IL-1β with Iba-1 provided additional evidence of enhanced microglial pyroptosis following RIR injury, which was suppressed by A151 treatment (Fig. 5H). Dual immunofluorescence staining further confirmed increased expression of pyroptosis markers (GSDMD, Caspase-1, and IL-1β) in retinal microglia during reperfusion. Taken together, these results indicate activation of the AIM2 inflammasome and pyroptosis of ischemic retinas after RIR injury, leading to the processing of inflammatory factors such as IL-1β. A151 effectively suppresses AIM2 activation and pyroptosis.

### Inhibition of cGAS–STING signaling protects the retina and RGC from ischemic injury

**Fig. 6:**
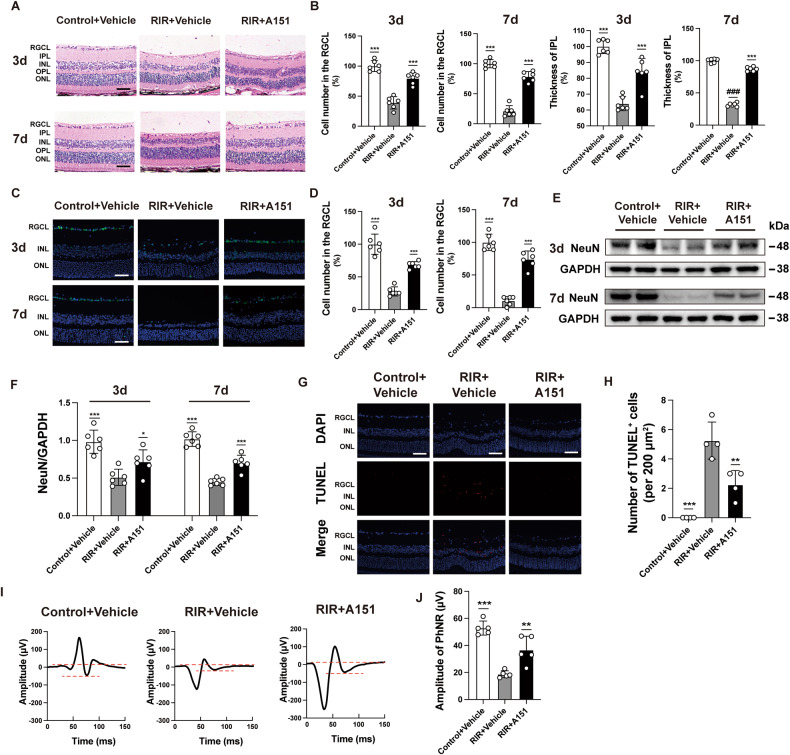
A151 protects the retina and improves RGC survival after RIR. **A**, **B** HE staining and quantitative analysis of RGC number and IPL thickness in mice in the various groups 3 d and 7 d after RIR injury. Scale bar: 50 μm (*n* = 6). **C** Representative images of RGCs in the mouse retinas in the indicated groups 3 d and 7 d after RIR injury. GRC cells were marked with NeuN. Scale bar: 50 μm. **D** Quantitative analysis of RGC (NeuN-positive) numbers in the retinas of the various groups 3 d and 7 d after RIR injury (*n* = 6). **E**, **F** Western blot and quantitative analyses of NeuN in the retinas of the various groups 3 d and 7 d after RIR injury (*n* = 6). **G** Representative images of TUNEL-positive cells in the retinas of the various groups 3 d after RIR injury. Scale bar: 50 μm. **H** quantification of TUNEL-positive cells in RGCL (*n* = 4). **I** Representative PhNR of retina after RIR injury in the various groups 7 d after RIR injury. **J** Quantification of the amplitude of PhNR in the various groups (*n* = 5). Data are shown as means ± SEM. The dots represent biological replicates. *P<0.05, ***P<0.001 compared with the RIR + vehicle group. One-way ANOVA followed by a Bonferroni post hoc test. RGCL retinal ganglion cell layer, IPL inner plexiform layer, INL inner nuclear layer, OPL outer plexiform layer, ONL outer nuclear layer.

In addition to the molecular findings showing that dsDNA-sensing cGAS in RIR was suppressed by A151 treatment, retinal morphology and RGC assessment allowed further analyses of the effects of the therapeutic intervention. Continuing in vivo observations supported the neuroprotective role of A151 in rescuing RGC loss 3 d after RIR injury, which persisted for 7 d, as measured by inner plexiform layer (IPL) thickness and the cell number in the RGCL after HE staining (Fig. 6A, B). This result suggested that inhibition of cytoplasm dsDNA-sensing cGAS by A151 could provide long-term benefits following RIR injury. The retinal immunofluorescent staining of NeuN also paralleled with the findings of the histological examination (Fig. 6C, D). Furthermore, quantitative analysis of NeuN was determined using Western blot analysis, and significant improvement in RGC survival was observed in the RIR + A151 group (Fig. 6E, F). In addition, TUNEL-positive cells in the retinal tissues further demonstrated a reduction in cell death after A151 treatment 3 d after RIR injury (Fig. 6G, H). We also examined the effect of A151 administration on RGC function. The PhNR amplitude was significantly lower in the RIR + A151 group than in the RIR + Vehicle group (Fig. 6I, J). Taken together, these data indicated that A151 inhibition of dsDNA-sensing cGAS protected mice from RIR injury.

### cGAS–STING signaling in retina samples taken from human end-stage glaucoma patients and healthy donors

**Fig. 7:**
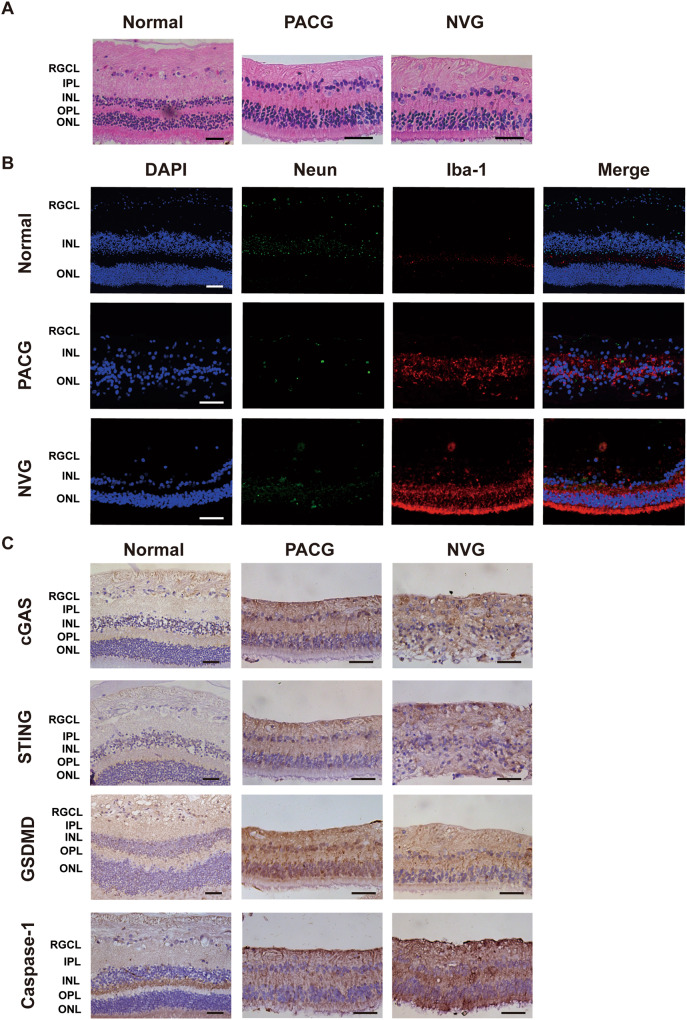
RGCs in retina samples taken from patients with end-stage glaucoma undergo cGAS activation. **A** HE staining of samples taken from a normal human donor and patients with primary angle-closure glaucoma (PACG) and neovascular glaucoma (NVG). Scale bar: 50 μm. **B** Representative immunofluorescent images of human retinal tissues. RGCs were marked with NeuN and microglia with Iba-1. Scale bar: 50 μm. **C** Immunohistochemical staining of cGAS, STING, GSDMD, and Caspase-1 expression in human retinal tissues. Scale bar: 50 μm. RGCL retinal ganglion cell layer, IPL inner plexiform layer, INL inner nuclear layer, OPL outer plexiform layer, ONL outer nuclear layer.

In this study, we found the increased expression of cGAS, STING, and the downstream proteins associated with neuroinflammation in RIR mice retinas, similarly, and that in end-stage glaucoma patients’ retinas. HE and immunofluorescent staining demonstrated that the RGC in end-stage glaucoma patients were almost completely lost, and the Iba-1 was upregulated (Fig. 7A, B). Notably, IHC showed that cGAS, STING, GSDMD, and Caspase-1 were significantly upregulated in the retinas of glaucoma patients (Fig. 7C). Thus, our findings suggest that the cGAS–STING pathway participates in the glaucomatous neuroinflammation and injury of RGC.

## Discussion

In the present study, we identified an important role of cytosolic dsDNA-sensing cGAS in RIR-induced neuroinflammation. Moreover, inhibitor A151 as a pharmacological intervention for antagonizing dsDNA cGAS and AIM2 attenuated the neuroinflammatory response by modulating microglia activation, pyroptosis and cytokines release. First, we demonstrated that dsDNA leaked from necrotic RGCs leads to the activation of microglia, and activated microglia significantly contribute to the pathophysiology of RGC death. Moreover, deletion or inhibition of cGAS in microglia significantly reduced microglia activation and inflammation response triggered by poly(dA:dT). Furthermore, our work demonstrated that A151 administration effectively attenuated microglia activation and subsequent RGC death by inhibiting the cGAS–STING pathway and pyroptosis-related molecules following RIR injury. Together, these findings suggested that inhibition of the dsDNA-sensing pathway may provide a potential therapeutic approach for reducing damage to RGC in acute glaucoma in a clinical setting.

Microglia are the primary resident immune cells in the CNS. Uncontrolled microglial cells contribute to sustained CNS injury that can threaten neuronal survival due to excessive inflammation [26]. The retina is an extension of the brain; therefore, similar inflammatory responses and ganglion cell injury may also occur in the retina [27]. Regarding the mechanisms of microglial neuroinflammation, recent studies have shown that microglia can sense cellular damage and stress by recognizing DAMPs via pattern recognition receptors. DAMPs include several molecules, some of which can be released from damaged cells [28]. The neuroinflammation itself is strictly associated with RGC dysfunction that triggers a vicious cycle: damaged RGCs induce microglia-dependent inflammation, which in turn further damages RGCs and initiates the subsequent release of DAMPs. It is well known that DNA can act as a DAMP to induce tissue toxicity and organ damage with transient elevation of IOP in a mouse model of acute glaucoma [29]. However, the precise effects of DAMP dsDNA and its relationship with microglial inflammation in patients with acute glaucoma remain under investigated. In this study, we found that neuroinflammation occurred soon after RIR injury, which directly contributed to RGC loss. The dsDNA released from damaged RGC following RIR injury is a specific DAMP recognized by microglia that triggers their activation and the release of inflammatory cytokines. This study revealed that the cytosolic release of dsDNA plays a primary role in retinal inflammation and degeneration.

cGAS signaling—an important innate immune pathway of cytosolic DNA sensing—has recently been investigated [30]. In this study, we observed the potent activation of cGAS in response to specific DAMP dsDNA elicited by retinal ischemic RGC death, which subsequently triggered downstream inflammatory events mediated by the STING cascade. By mimicking an in vitro model of dsDNA recognition following RIR injury, our study showed that activated microglia initiate the cGAS–STING pathway, thus promoting inflammation cytokine secretion. Deletion or inhibition of cGAS in microglia effectively decreased microglial activation and attenuated the inflammatory response. These findings imply that the activated microglia-triggered cGAS–STING pathway contributes to the ischemia-induced death of RGC. In an in vivo study, we found that the recognition of DAMP dsDNA by cGAS following RIR injury caused the activation of NF-κB and stimulated the IFN pathway. Neuroinflammation can result in RGC death and retinal damage, leading to the release of DAMPs that can, in turn, activate a further inflammation response. The resulting significant release of inflammatory cytokines propagates a vicious cycle of inflammation that plays a critical role in the progression of retinal damage. Thus, breaking this cycle may be the key to preventing irreversible injury to RGC in acute glaucoma and improving the prognosis following RIR injury. Studies on the intraperitoneal (IP) administration of ODNs have shown the protective role of their anti-inflammatory properties in the brains of ischemic stroke and Alzheimer’s disease patients [18, 31, 32], suggesting that synthetic oligonucleotides may enter the brain parenchyma through the blood–brain barrier (BBB) and play a role in the CNS. Previous studies have also shown that IP injection of suppressive ODN A151 has the capacity to pass the blood–retina barrier and protect the retina [33]. Microglia polarization has been proven to play pivotal roles in regulating inflammatory reactions [25]. In vitro study indicated that A151 could efficiently switch microglia from the M1 to M2 phenotype in an inflammation-inducing environment. Our study further confirmed the ability of A151 to effectively inhibit the activation of cGAS and STING, subsequently inhibiting the transcription factors NF-κB and IRF3. Using A151 to disrupt this cycle can effectively improve the inflammatory milieu of the retina and reduce inflammation-associated neuronal damage, preventing the loss of RGCs. These findings suggest that systemic delivery of A151, absorbed from the blood into the retina, could provide novel evidence that inhibition of the cGAS–STING pathway can reduce RIR injury and improve outcomes.

AIM2 activation initiates inflammasome assembly—an innate immune complex that further activates inflammatory cascades. This process is directly stimulated by cytosolic dsDNA and its sensor cGAS–STING pathway. This induces procaspase-1 cleavage, accompanied by the maturation and secretion of cytokines IL-18 and IL-1β, leading to secondary inflammatory cascades and neuronal cell death in the CNS [22, 34]. Nevertheless, there is currently an absence of specific evidence for a relationship between the AIM2 inflammasome and acute glaucoma. In this study, our results showed significantly higher expression of AIM2, ASC, Caspase-1, GSDMD, and IL-1β in the retinas of mice 3 d after RIR injury compared to the control group, indicating that AIM2 activation is probably involved in the development of acute glaucoma. In this study, colocalization of GSDMD, caspsase-1, and IL-1β mainly with Iba-1 in the ischemic retinas suggested that microglia were the main sources of activated inflammasomes following RIR injury. For the first time, our study provides evidence for the efficacy of pharmacological inhibition of the AIM2 inflammasome in the context of RIR injury in vivo, indicating that A151 plays a crucial role in the regulation of high-IOP-induced retinal ischemic RIR injury and microglial pyroptosis. Thus, regulation of cGAS on microglia is perhaps the main but not the exclusive mechanism responsible for the protective effect of A151. The other potential molecular mechanisms and interrelationships between pyroptosis and the cGAS–STING pathway, as well as how they work together to induce RGC death, require further investigation.

Overall, our study revealed that cytosolic dsDNA is involved in the progression of RGC death in acute glaucoma. We demonstrated for the first time that dsDNA-sensing cGAS can mediate the neuroinflammation associated with retinal injury and RGC death following RIR injury. Moreover, pharmacological interventions aimed at using the inhibitor A151 to antagonize dsDNA cGAS can suppress the neuroinflammatory response by inhibiting the cGAS–STING pathway, preventing microglial pyroptosis, and thus significantly attenuating retinal injury and reducing RGC death following RIR. Our study provides novel insights into the role of neuroinflammation in elevated IOP-induced retinal ischemic injury and suggests that targeting dsDNA-sensing cGAS may be a promising strategy for innovative treatment of acute glaucoma.

## Methods

### Animals and treatment

Male C57BL/6J mice (6–8 weeks old) were purchased from Charles River Laboratories (Beijing, China) and housed in standard cages in a pathogen-free facility on a 12-h light/dark cycle with free access to food and water. The RIR animal model was established by inducing acute intraocular hypertension, as previously reported [7]. In brief, the mice were anesthetized with sodium pentobarbital (100 mg/kg) by IP injection and 0.5% proparacaine (Alcon, Texas, USA) was topically applied to the left eyes. The corneas were treated with 1% tropicamide (Santen, Osaka, Japan) to dilate the pupils. The anterior chambers (ACs) were cannulated with a 33-gauge needle attached to a normal saline reservoir, elevated to 150 cm, and continued for 90 min. This operation maintained an average IOP of 110 mmHg, according to our previous study [35]. The sham operation, which served as the control, was performed without elevating the IOP. Withdrawal of the needle allowed the IOP to return to normal. Levofloxacin eye drops (Santen, Osaka, Japan) were applied to the treated eyes to prevent infection. Eyes with cannulation-induced cataracts, iris injury/bleeding, or AC leakage were excluded.

Synthetic oligonucleotide A151 (5^’^-TTAGGGTTAGGGTTAGGGTTAGGG-3’) was synthesized with a phosphorothioate backbone, unless otherwise specified, at Tsingke Biotechnology Co., Ltd. (Beijing, China). Poly(dA:dT) (Cat tlrl-patn) and ODN TTAGGG (ODN A151) were purchased from InvivoGen. A151 or its vehicle was administered by IP injection at 300 μg in 0.2-ml phosphate-buffered saline (PBS) [33] per mouse immediately after reperfusion, followed by daily injections until the third postoperative day. The mice with RIR injuries were randomly assigned to treatment groups with A151 or a vehicle, and control mice were injected with the same volumes of PBS.

### Histology

After the mice were sacrificed with deep anesthesia, eyes were dissected at designated time points, fixed in FAS eye fixation solution (HaoKe Bio, Hangzhou, China), dehydrated using an increasing ethanol gradient, and embedded in paraffin as previously described [36]. Sections (thickness 5 μm) across the optic nerve of each eye were prepared and stained with HE. Four observational areas (200 × 200 μm^2^) of each retina were selected 1 mm from the optic disc and all the measurements were repeated by two blinded investigators. The IPL thickness and the number of cells in the RGCL were quantified using ImageJ software [35]. Major visceral organs, including heart, liver, spleen, lung, and kidney, were fixed in 4% paraformaldehyde (Biosharp, Guangzhou, China) and stained with HE for histological observation.

### IHC

For IHC, sections were routinely dewaxed and hydrated, and the antigen was retrieved by incubation at 95 °C in 10 mM sodium citrate buffer for 30 min. Thereafter, the slides were immersed in 3% H_2_O_2_ for 10 min at room temperature (RT) in the dark and blocked with normal goat serum (NGS) solution at RT for 1 h. The slides were then immunostained with antibodies (anti-GSDMD, Caspase-1, cGAS, and STING; Supplementary Table S1) at 4 °C overnight. The sections were washed with PBS, followed by incubation with horseradish peroxidase (HRP)–conjugated goat anti-rabbit IgG polyclonal antibodies at RT for 23 min. Subsequently, the sections were stained with 3,3’-diaminobenzidine and counterstained with hematoxylin. All slides were evaluated and photographed using a Leica DM2500 microscope and quantified with ImageJ software.

### Immunofluorescence staining analysis

The frozen sections (8 μm) or BV2 cells cultured on coverslips (20 mm) were fixed with 4% PFA for 20 min at RT and permeabilized with 0.3% Triton X-100 in PBS for 1 h. After washing in PBS, retinal sections were blocked in buffer (5% NGS, 2% BSA, and 0.1% Tween-20 in PBS) for 1 h, then incubated overnight with primary antibodies (anti-dsDNA, Iba-1, NeuN, STING, 53BP1, Caspase-1, and GSDMD; Supplementary Table S1) at 4 °C overnight, followed by 1 h of incubation with the species-specific secondary fluorescent antibodies. Nuclei were contained with DAPI Fluoromount-G^TM^ (Yeasen Biotech, Shanghai, China). All slides were imaged using a Leica DMi8 microscope and quantified with ImageJ software.

### Quantitative real-time polymerase chain reaction (qPCR) analysis

Total RNAs from mouse retinal tissues were isolated using TRIzol reagent (Invitrogen, California, USA) and quantified using a NanoDrop™ 2000 Spectrophotometer (Thermo Fisher Scientific, Massachusetts, USA). cDNA was synthesized with a PrimeScript™ RT Reagent Kit (Takara, Kyoto, Japan). Thereafter, qPCR was performed using a 7500 Fast Real-Time PCR system (Thermo Fisher Scientific, Massachusetts, USA) with the SYBR PrimeScript™ Plus RT-PCR Kit (Takara, Kyoto, Japan). Gene expression was normalized to GAPDH mRNA. The fold change in gene expression was calculated by comparing it with a standard cycle. The primers of the target genes are listed in Supplementary Table S2.

### Western blot analysis

Total proteins of retinal tissue or cultured cells were extracted using a protein extraction kit (BC3710, Solarbio, Beijing, China). The protein concentration was determined with a BCA Protein Assay Kit (Thermo Fisher Scientific, Massachusetts, USA) according to the manufacturer’s instructions. Equal amounts of protein (25–35 μg) were loaded onto SDS-PAGE gels (ACE Biotechnology, Nanjing, China) and transferred to PVDF membranes (Millipore, New Jersey, USA). The membranes were blocked in blocking buffer (EpiZyme, Shanghai, China) and incubated with the corresponding primary antibodies (Supplementary Table S1) overnight at 4 °C. The membranes were then incubated for 1 h at RT with the appropriate secondary antibodies conjugated to HRP, after which the blots were visualized with an enhanced chemiluminescence (ECL) kit (Biosharp, Guangzhou, China) and recorded with a ChemiDoc™ imaging system (Bio-Rad Laboratories, California, USA). The bands were quantified by densitometry using ImageJ software.

### TUNEL assay

Terminal deoxynucleotidyl transferase (TdT)-mediated dUTP nick end labeling (TUNEL) assay was performed using paraffin section following the One Step TUNEL Apoptosis Assay Kit method (Beyotime, Jiangsu, China). The sections were stained with DAPI and observed under a Leica DMi8 microscope and quantified with ImageJ software.

### Electroretinogram (ERG)

ERG was recorded using Ganzfeld Q450 (Roland, Brandenburg, Germany). RGC function was evaluated by measuring the amplitude of Photopic Negative Response (PhNR) [37]. Briefly, the mice were anesthetized by IP injection of sodium pentobarbital (100 mg/kg) followed by eye dropping 1% tropicamide (Santen, Osaka, Japan) for pupillary dilation, 0.5% proparacaine (Alcon, Texas, USA) for corneal anesthesia and carbomer gel for eye lubrication. Subsequently, the reference electrodes were fixed under the skin of cheeks and the ground electrode was fixed under the skin near the tail, while the recording electrode were placed on the center of corneas. For assessment of PhNR, light stimulation was performed at 0.4 cd seconds per meter squared (cd.s/m^2^) red light against a blue background of 25 cd.s/m^2^ for 2 ms with inter-flash interval for 0.5 s. The PhNR amplitude was defined as the peak of the negative wave following the b-wave that was measured relative to the baseline.

### Cell culture and treatment

The BV2 microglial cell line was provided by Professor Chen Gao (The Second Affiliated Hospital of Zhejiang University). The cells were maintained in Dulbecco’s modified Eagle medium (DMEM, high glucose; Gibco, California, USA) supplemented with 10% fetal bovine serum (FBS; AusgeneX, Queensland, Australia) and 1% penicillin-streptomycin (Gibco, California, USA) solution at 37 °C in a humidified atmosphere at 5% CO_2_. BV2 cells were cultured in 6-well plates to 70% confluence and primed for 3 h with 500 ng/ml lipopolysaccharide (LPS) (Solarbio, Beijing, China), then predissolved in PBS before exposure to poly(dA:dT) (2 μg/ml) with or without ODN A151 (1 h pretreatment; 3 μM). The synthetic DNA analogue poly(dA:dT) (InvivoGen, California, USA) was transfected with Lipofectamine™ 2000 (Invitrogen, California, USA), according to the manufacturer’s protocol, in OptiMEM™ (Gibco, California, USA) for 6 h [18, 23]. The cells were transfected using Lipofectamine™ RNAiMAX (Invitrogen, California, USA) according to the manufacturer’s protocol. The cells were transfected with negative control (NC) small interfering RNAs (siRNAs) and siRNAs (100 μM) targeting cGAS and STING 12 h before being transfected with poly(dA:dT). siRNAs targeting mouse cGAS or STING were designed and synthesized by Tsingke Biotechnology Co., Ltd., and NC siRNAs were purchased from this company. The sequences of the siRNAs were as follows: siGAS (GAUUGAAACGCAAAGAUAUTT) and siSTING (GCUAUGAUUCUACUAUCGUTT).

### CCK-8 assay

Cell viability was determined by the CCK-8 (Dojindo, Kumamoto, Japan) assay. Brifly, the BV2 cells were plated at a seeding density of 1 × 10^4^ cells/well in a 96‐well plate and incubated overnight. Then the cells were treated with PBS or A151 (3 μM) for 24 h. Subsequently, 10 μL CCK-8 solution (10 μl per 100 μl of medium in each well) was added and incubated at 37 °C for 2 h. The absorbance was measured at 450 nm using an absorbance microplate reader (Bio-Rad Laboratories, California, USA).

### Live/dead assay

For Calcein-AM/ PI assay (Yeasen Biotech, Shanghai, China), BV2 cells were seeded at a density of 1.5 × 10^5^ cells/well in a 12-well plate and incubated overnight. Subsequently, the cells were treated with PBS or A151 (3 μM) for 24 h. After that, the cells were rinsed three times with PBS and stained with Calcein-AM (0.67 μM) and PI (1.5 μM) for 15 min at 37 °C. Finally, the live (green) or dead (red) cells were observed and photographed with fluorescence microscope (Leica DMi8, Germany).

### Human eye tissue

Human retinas were obtained from patients with end-stage glaucoma and a deceased healthy donor during eye enucleation. Retinas were fixed, dehydrated, embedded, and sectioned in the same way as the mice retinas. HE staining, immunofluorescent staining, and immunohistochemistry were performed on samples of human retinas. All human retinal tissues were obtained from the Ophthalmology Department of the Second Affiliated Hospital of Zhejiang University between February 2021 and September 2021. The details of the clinical information are shown in Supplementary Table S3.

### Statistics

All data are reported as means ± standard deviation (SD). One-way analysis of variance (ANOVA) followed by a Bonferroni post hoc test were used to compare differences among more than two groups, and a two-tailed unpaired Student’s *t* test was used to assess differences between two groups using GraphPad Prism version 9.0 software (GraphPad Software, Inc.). *P* values less than 0.05 indicated statistical significance.

### Supplementary information

Supplemental Material

Checklist

## Data Availability

All data generated or analyzed during this study are included in this published article and its Additional files.
