# Supplementary material for: Inhibition of cGAS–STING pathway alleviates neuroinflammation-induced retinal ganglion cell death after ischemia/reperfusion injury
Source: Cell Death Dis. 2023 Sep 19;14(9):615. doi: 10.1038/s41419-023-06140-0 (PMC10509212; doi:10.1038/s41419-023-06140-0)
Supplement: Supplementary file 1 — Supplemental Material [file 41419_2023_6140_MOESM1_ESM.docx]

**Supplementary information for**

**Inhibition of cGAS–STING pathway alleviates** **neuroinflammation–induced** **retinal ganglion cell death after ischemia/reperfusion injury**

**This file includes：**

Supplementary Figure S1 to S5

Supplementary Table S1 to S3

**Supplemental Figures**


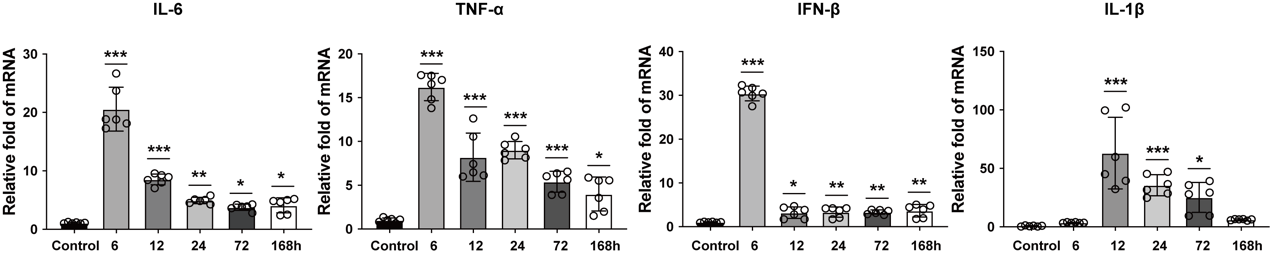


**Fig. S1 Expression changes in inflammatory cytokines in normal or IR-injured retinas**

The mRNA expression of inflammatory cytokines, including IL-6, TNF-α, IFN-β, and IL-1β, at different times following IR injury (n = 6). Data are shown as means ± SEM. The dots represent biological replicates. **p* < .05, ***p* < .01, ****p* < .001. One-way ANOVA followed by a Bonferroni post hoc test.


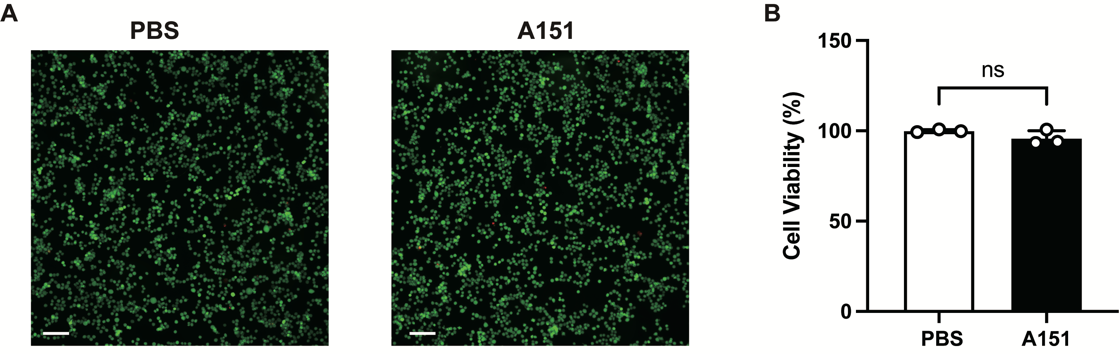


**Fig. S2 In Vitro Cytotoxicity Assessment**

**A** The live/dead (Calcein-AM/PI) assay of the BV2 cells treated with PBS or A151. Scale bar: 50 μm. **B** The CCK-8 assay of the BV2 cells treated with PBS or A151 (n = 3). Data are shown as means ± SEM. The dots represent biological replicates. A two-tailed Student’s t-test.


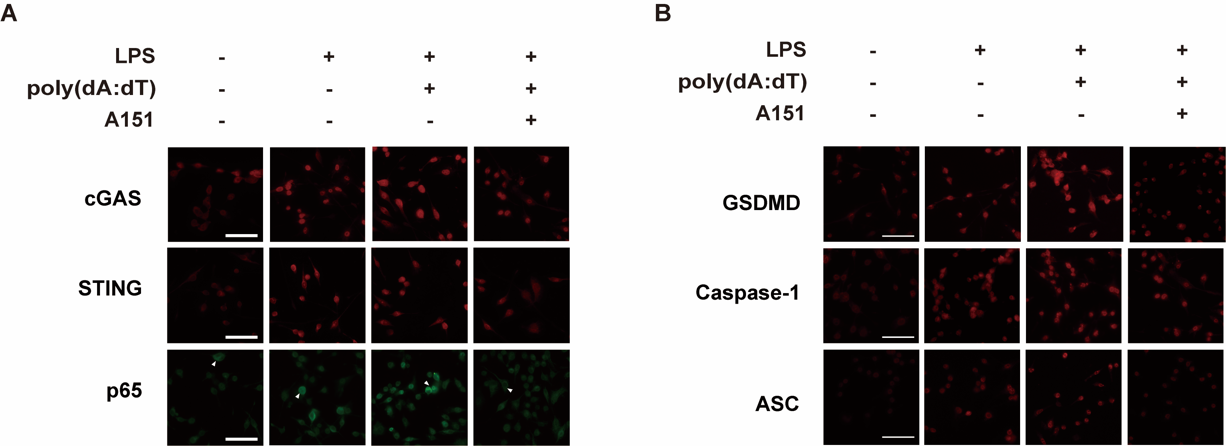


**Fig. S3** **Expression changes of cGAS-STING pathway and pyroptosis-associated proteins after A151 treatment in BV2 cells**

**A, B** Immunofluorescence was conducted to detect cGAS, STING, and NF-κB expression levels (arrows indicate NF-κB translocation), and pyroptosis-associated molecules (GSDMD, caspase-1, and ASC) in BV2 cells. Scale bar: 50 μm.


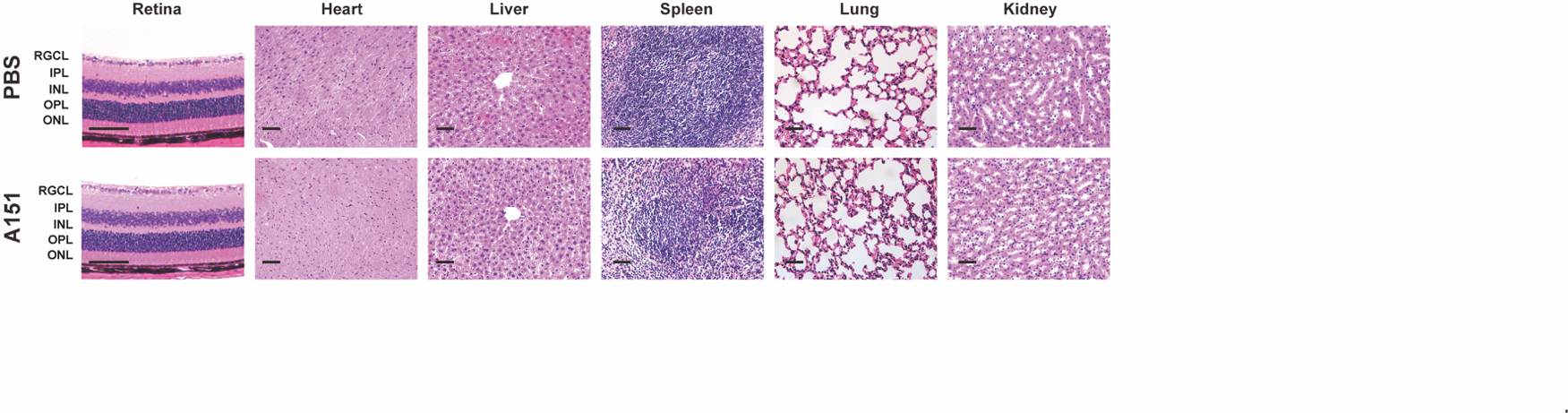


**Fig. S4 In Vivo Biocompatibility Assessment in Healthy Mice**

HE staining of the retina and main visceral organs following 3 days treatment of PBS or A151 in healthy mice. Scale bar:100 μm. RGCL = retinal ganglion cell layer, IPL = inner plexiform layer, INL = inner nuclear layer, OPL = outer plexiform layer, ONL = outer nuclear layer.


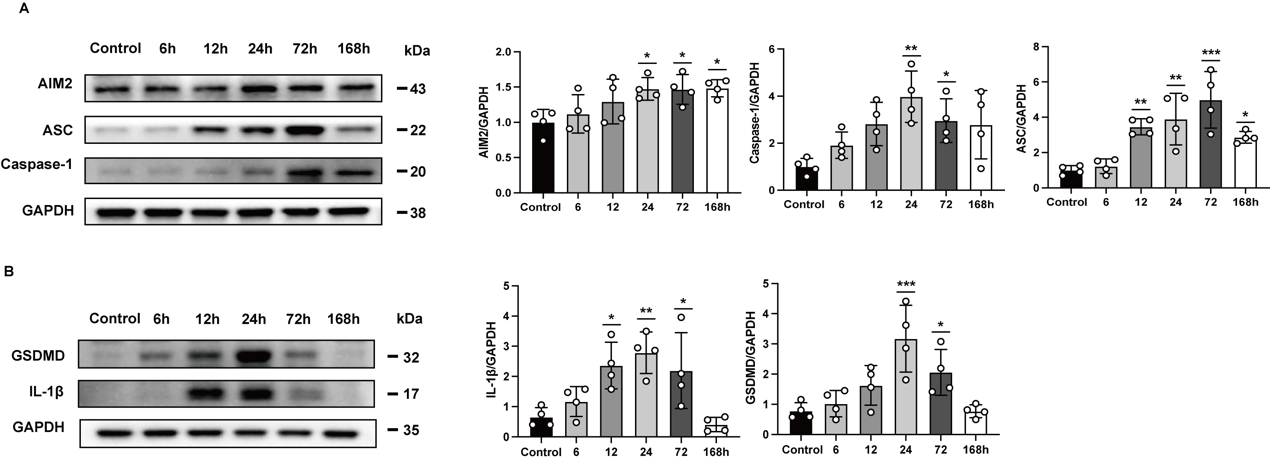


**Fig. S5 Expression changes in the AIM2 inflammasome in normal or IR-injured retinas**

**A** Western blot and quantitative analyses of AIM2, ASC, and Caspase-1 in the retinas at different times after IR injury (n = 4). **B** Western blot and quantitative analyses of GSDMD and IL-1β in the retinas at different times after IR injury (n = 4). Data are shown as means ± SEM. The dots represent biological replicates. **p* < .05, ***p* < .01, ****p* < .001. One-way ANOVA followed by a Bonferroni post hoc test.

**Supplemental Tables**

**Table S1. antibodies used for medication, immunofluorescent staining and immunohistochemistry, and Western blot.**

| Primary Antibodies | Source | Company | Catalog No. | Dilution^1^ |
| --- | --- | --- | --- | --- |
| cGAS | Rabbit mAb | Cell Signaling Technology | 31659S | 1:1000 |
| TBK | Rabbit mAb | Cell Signaling Technology | 3504S | 1:1000 |
| Phospho--TBK | Rabbit mAb | Cell Signaling Technology | 5483S | 1:1000 |
| IRF3 | Rabbit mAb | Cell Signaling Technology | 4302S | 1:1000 |
| Phospho--IRF3 | Rabbit mAb | Cell Signaling Technology | 29047S | 1:1000 |
| NF-κB p65 | Rabbit mAb | Cell Signaling Technology | 8242S | 1:1000  1:500 IF |
| Phospho-NF-κB p65 | Rabbit mAb | Cell Signaling Technology | 3033S | 1:1000 |
| AIM2 | Rabbit mAb | Cell Signaling Technology | 63660S | 1:1000 |
| ASC | Rabbit mAb | Cell Signaling Technology | 67824S | 1:1000 |
| IL-1β | Goat pAb | R&D Systems | AF-401-NA | 1:800 |
| Caspase-1 | Rabbit pAb | ABclonal | A0964 | 1:500 |
| GSDMD | Rabbit pAb | ABclonal | A20197 | 1:500 |
| cGAS | Rabbit mAb | ABclonal | A8335 | 1:200 IHC-P |
| Iba-1 | Rabbit mAb | Abcam | ab178846 | 1:500 IF |
| NeuN | Rabbit mAb | Abcam | ab177487 | 1:1000  1:100 IF |
| 53BP1 | Rabbit mAb | Abcam | Ab175933 | 1:100 IF |
| GFAP | Rabbit mAb | Abcam | Ab7260 | 1:1000 IF |
| dsDNA | Mouse mAb | Santa cruz | sc58749 | 1:50 IF |
| IL-1β | Mouse mAb | Santa cruz | sc52012 | 1:50 IF |
| GSDMD | Mouse pAb | Santa cruz | sc393581 | 1:50 IF |
| Caspase-1 | Mouse pAb | Santa cruz | sc392736 | 1:50 IF |
| GSDMD | Rabbit pAb | Proteintech | 20770-1-AP | 1:100 IHC-P |
| STING | Rabbit pAb | Proteintech | 19851-1-AP | 1:1000  1:50 IF  1:2000 IHC-P |
| Caspase-1 | Rabbit pAb | Proteintech | sc392736 | 1:100 IHC-P |
| HRP-conjugated GAPDH | Mouse mAb | Proteintech | HRP-60004 | 1:10000 |
| Secondary Antibodies | **Source** | **Company** | **Catalog No.** | **Dilution^1^** |
| Goat Anti-Rabbit IgG (H&L) (HRP) | Goat pAb | Proteintech | SA00001-15 | 1:10000 |
| Rabbit anti-Goat IgG (H&L) (HRP) | Rabbit pAb | Proteintech | SA00001-4 | 1:10000 |
| Goat anti-Rabbit IgG (H&L) (Alexa Fluor® 555) | Goat pAb | Cell Signaling Technology | 4413 | 1:1000 IF |
| Goat anti-Rabbit IgG (H&L) (Alexa Fluor® 488) | Goat pAb | Cell Signaling Technology | 4412 | 1:1000 IF |
| Goat anti-Rabbit IgG (H&L) (HRP) | Goat pAb | Solarbio | SE134 | 1:200 IHC-P |
| Goat anti-mouse IgG (H&L) (Alexa Fluor® 488) | Goat pAb | Cell Signaling Technology | 4408S | 1:1000 IF |
| Goat anti-mouse IgG (H&L) (Alexa Fluor® 555) | Goat pAb | Cell Signaling Technology | 4409S | 1:1000 IF |

^1^The dilution ratio of antibodies without following IF or IHC-P was used for the Western blot.

**Table S2. Gene-specific primers (5’-3’, F = Forward, R = Reverse).**

| **Gene** | **Primer** |
| --- | --- |
| cGAS -F | GTTCAAACACAAGAAATGCACTG |
| cGAS -R | GCTGACGGAGTACACAATCCT |
| STING -F | TGAAAGGCTCTTCATTGTCTCTT |
| STING -R | TGGCATCTTCTGCTTCCTAGA |
| IL-6 -F | TCCAGTTGCCTTCTTGGGAC |
| IL-6 -R | GTGTAATTAAGCCTCCGACTTG |
| IL-1β-F | GCAACTGTTCCTGAACTCAACT |
| IL-1β-R | ATCTTTTGGGGTCCGTCAACT |
| TNFα-F | CCTCTCATGCACCACCATCA |
| TNFα-R | GCATTGCACCTCAGGGAAGA |
| Ifnβ-F | GCCTTTGCCATCCAAGAGATGC |
| Ifnβ-R | ACACTGTCTGCTGGTGGAGTTC |
| IL18 -F | ACTTTGGCCGACTTCACTGT |
| IL18 -R | GGGTTCACTGGCACTTTGAT |
| GAPDH -F | GCCACCCAGAAGACTGTGGAT |
| GAPDH -R | GGAAGGCCATGCCAGTGA |

**Table S3. Clinical information of the end-stage glaucoma patients and healthy donor.**

| **No.** | **Age** | **Gender** | **Patient type** | **Visual Acuity** | **Latest IOP (mmHg)^1^** |
| --- | --- | --- | --- | --- | --- |
| 1 | 25 | Male | Healthy Donor | N/A | 17.0 |
| 2 | 69 | Female | Primary Angle closure Glaucoma | NLP | 53.0 |
| 3 | 45 | Male | Neovascular Glaucoma | NLP | 55.0 |

^1^Normal range: 10–21 mmHg.

NLP: No Light Perception. N/A: Not Applicable. IOP: Intraocular Pressure.
